# Update: Influenza Activity — United States, September 30, 2018–February 2, 2019

**DOI:** 10.15585/mmwr.mm6806a1

**Published:** 2019-02-15

**Authors:** Lenee Blanton, Vivien G. Dugan, Anwar Isa Abd Elal, Noreen Alabi, John Barnes, Lynnette Brammer, Alicia P. Budd, Erin Burns, Charisse N. Cummings, Shikha Garg, Rebecca Garten, Larisa Gubareva, Krista Kniss, Natalie Kramer, Alissa O’Halloran, Carrie Reed, Melissa Rolfes, Wendy Sessions, Calli Taylor, Xiyan Xu, Alicia M. Fry, David E. Wentworth, Jacqueline Katz, Daniel Jernigan

**Affiliations:** 1 Influenza Division, National Center for Immunization and Respiratory Diseases, CDC.

CDC collects, compiles, and analyzes data on influenza activity and viruses in the United States. During September 30, 2018–February 2, 2019,* influenza activity^†^ in the United States was low during October and November, increased in late December, and remained elevated through early February. As of February 2, 2019, this has been a low-severity influenza season (*1*), with a lower percentage of outpatient visits for influenza-like illness (ILI), lower rates of hospitalization, and fewer deaths attributed to pneumonia and influenza, compared with recent seasons. Influenza-associated hospitalization rates among children are similar to those observed in influenza A(H1N1)pdm09 predominant seasons; 28 influenza-associated pediatric deaths occurring during the 2018–19 season have been reported to CDC. Whereas influenza A(H1N1)pdm09 viruses predominated in most areas of the country, influenza A(H3N2) viruses have predominated in the southeastern United States, and in recent weeks accounted for a growing proportion of influenza viruses detected in several other regions. Small numbers of influenza B viruses (<3% of all influenza-positive tests performed by public health laboratories) also were reported. The majority of the influenza viruses characterized antigenically are similar to the cell culture–propagated reference viruses representing the 2018–19 Northern Hemisphere influenza vaccine viruses. Health care providers should continue to offer and encourage vaccination to all unvaccinated persons aged ≥6 months as long as influenza viruses are circulating. Finally, regardless of vaccination status, it is important that persons with confirmed or suspected influenza who have severe, complicated, or progressive illness; who require hospitalization; or who are at high risk for influenza complications be treated with antiviral medications.

## Virus Surveillance

**FIGURE 1:**
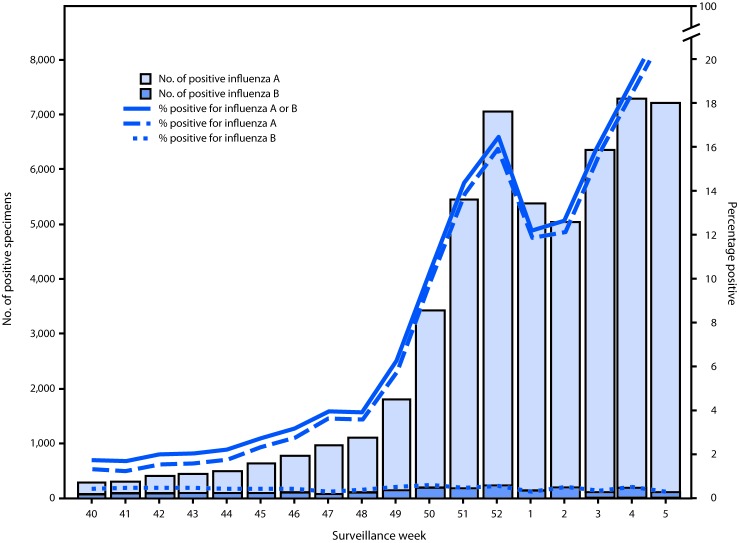
Number* and percentage of respiratory specimens testing positive for influenza reported by clinical laboratories, by influenza virus type and surveillance week – United States, September 30, 2018–February 2, 2019^†^ * Results for 54,381 (10.1%) of 536,301 specimens tested were positive during September 30, 2018–February 2, 2019. ^†^ As of February 2, 2019.

U.S. World Health Organization (WHO) and National Respiratory and Enteric Virus Surveillance System laboratories, which include both clinical and public health laboratories throughout the United States, contribute to virologic surveillance for influenza. During September 30, 2018–February 2, 2019, clinical laboratories tested 536,301 specimens for influenza virus; among these, 54,381 (10.1%) tested positive, including 52,028 (95.7%) for influenza A and 2,353 (4.3%) for influenza B (Figure 1). The percentage of specimens testing positive for influenza each week ranged from 1.7% to 21.6%.

**FIGURE 2:**
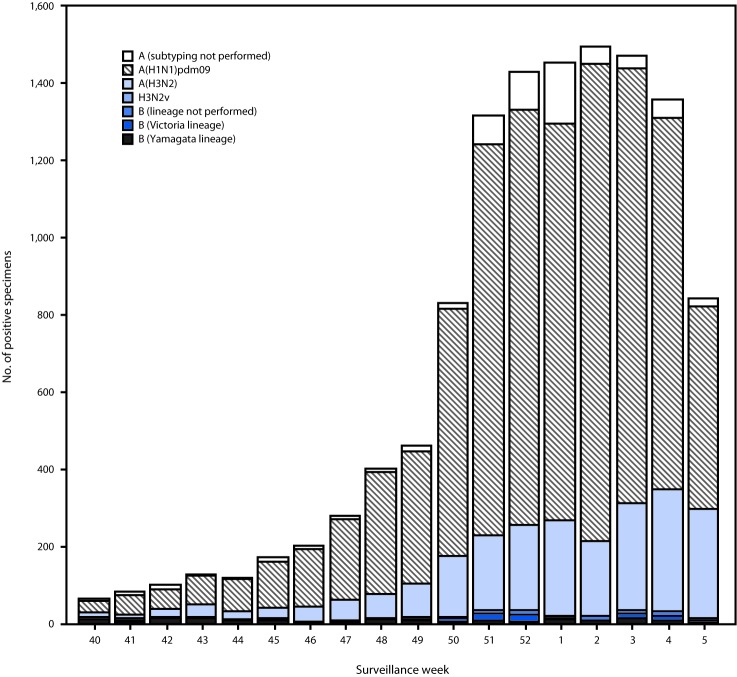
Number* of respiratory specimens testing positive for influenza reported by public health laboratories, by influenza virus type, subtype/lineage, and surveillance week – United States, September 30, 2018–February 2, 2019^†^ * N = 12,200. ^†^ As of February 2, 2019.

Public health laboratories tested 30,344 specimens during September 30, 2018–February 2, 2019; 12,200 were positive for influenza viruses, including 11,863 (97.2%) positive for influenza A and 337 (2.8%) for influenza B (Figure 2). Among the 11,284 influenza A viruses subtyped, 9,023 (80.0%) were influenza A(H1N1)pdm09, and 2,261 (20.0%) were influenza A(H3N2). Influenza B lineage information was available for 249 (73.9%) influenza B viruses; 143 (57.4%) were B/Yamagata lineage, and 106 (42.6%) were B/Victoria lineage. Influenza A(H1N1)pdm09 viruses accounted for the majority of circulating viruses; however, in the southeastern United States, influenza A(H3N2) viruses have predominated (accounting for 29.8% of all influenza A(H3N2) viruses reported in the United States). From late December 2018 to early February 2019, influenza A(H3N2) viruses have accounted for a growing proportion of influenza viruses detected in several other regions.

Among 10,766 (88.2%) patients with positive test results for seasonal influenza virus by public health laboratories and for whom age data were available, 1,627 (15.1%) were aged 0–4 years; 3,493 (32.4%) were aged 5–24 years; 3,991 (37.1%) were aged 25–64 years; and 1,654 (15.4%) were aged ≥65 years. Influenza A(H1N1)pdm09 viruses predominated among all age groups, ranging from 64.4% among persons aged ≥65 years to 79.4% among persons aged 25–64 years. The percentage of influenza A(H3N2) viruses ranged from 30.1% among persons aged ≥65 years to 13.5% in persons aged 25–64 years. From late December 2018 to early February 2019, the proportion of influenza A(H3N2) viruses among persons aged 5–24 years has increased from 24.4% to 46.2%. Among all age groups, influenza B viruses have accounted for ≤5% of positive influenza test results.

## Antigenic and Genetic Characterization of Influenza Viruses

In the United States, public health laboratories participating in influenza surveillance as WHO collaborating laboratories are asked to submit a subset of influenza-positive respiratory specimens to CDC for virus characterization according to specific guidelines.^§^ Data obtained from antigenic characterization are important in the assessment of the similarity between reference vaccine viruses and circulating viruses. In vitro antigenic characterization data acquired through hemagglutination inhibition assays or virus neutralization–based focus reduction assays evaluate whether genetic changes in circulating viruses affect antigenicity; substantial differences could affect vaccine effectiveness. Nearly all influenza viruses received by CDC are genomically characterized using next generation sequencing, and the genomic data are analyzed and submitted to public databases (GenBank: https://www.ncbi.nlm.nih.gov/genbank or EpiFlu: https://www.gisaid.org/). CDC has genetically characterized 769 influenza viruses collected and submitted by U.S. laboratories since September 30, 2018, including 450 influenza A(H1N1)pdm09 viruses, 239 influenza A(H3N2) viruses, and 80 influenza B viruses. A subset of these viruses were also antigenically characterized.

Phylogenetic analysis of the hemagglutinin (HA) gene segments from the 450 characterized A(H1N1)pdm09 viruses determined that all belonged to clade 6B.1. Considerable genetic diversity within clade 6B.1 has emerged; further evolution in the HA gene has occurred, resulting in the circulation of multiple clades. Among 194 A(H1N1)pdm09 viruses antigenically characterized, 191 (98.5%) were antigenically similar (analyzed using hemagglutination inhibition with ferret antisera) to A/Michigan/45/2015 (6B.1), a cell culture–propagated A/Michigan/45/2015-like reference virus representing the A(H1N1)pdm09 component for the 2018–19 Northern Hemisphere influenza vaccines.

A total of 239 influenza A(H3N2) viruses were sequenced, and phylogenetic analysis of the HA gene segments illustrated that multiple clades/subclades were cocirculating. Circulating viruses possessed HA gene segments that belonged to clade 3C.2a (55; 23.0%), subclade 3C.2a1 (98; 41.0%), or clade 3C.3a (86; 36.0%). The frequency of 3C.3a viruses has increased, from 16% of the A(H3N2) viruses sequenced and collected in November 2018 to 51% of those sequenced and collected in December 2018. The geographic distribution of 3C.3a viruses also has increased, from the southeastern United States in November 2018 to throughout the continental United States by the end of December 2018. Among the 145 representative A(H3N2) viruses that were antigenically characterized by focus reduction assay with ferret antisera, 102 (70.3%) were well-inhibited (reacting at titers that were within fourfold of the homologous virus titer) by ferret antisera raised against A/Singapore/INFIMH-16–0019/2016 (3C.2a1), a cell culture–propagated reference virus representing the A(H3N2) component of 2018–19 Northern Hemisphere influenza vaccines. Forty-three (29.7%) viruses reacted poorly (at titers that were reduced eightfold or more when compared with the homologous virus A/Singapore/INFIMH-16–0019/2016) and, of the 43 viruses, 42 (97.7%) belonged to clade 3C.3a. However, only 28 of the 145 viruses tested were well-inhibited by antiserum raised against egg-propagated A/Singapore/INFIMH-16–0019/2016 reference virus representing the A(H3N2) vaccine component, likely because of egg-adaptive amino acid changes in the HA of the egg-propagated virus.

Among influenza B viruses, phylogenetic analysis of 50 influenza B/Yamagata lineage viruses determined that the HA gene segments belonged to clade Y3. Thirty-three B/Yamagata lineage viruses were antigenically characterized, and all were antigenically similar to cell culture–propagated B/Phuket/3073/2013, the reference virus representing the B/Yamagata lineage component of quadrivalent vaccines for the 2018–19 Northern Hemisphere influenza season.

Among the 30 influenza B/Victoria lineage viruses sequenced and phylogenetically analyzed, the HA gene segment of all viruses belonged to genetic clade V1A (10; 33.3%), subclade V1A.1 (18; 60.0%), or subclade V1A-3Del (2; 7%). Viruses with a two-amino acid–deletion (162–163) in the HA protein belong to subclade V1A.1, and viruses with a three-amino acid–deletion (162–164) in the HA protein belong to subclade V1A-3Del. Twenty-one B/Victoria lineage viruses were antigenically characterized and 15 (71.4%) were antigenically similar to cell culture–propagated B/Colorado/06/2017-like V1A.1 reference virus. Six (28.6%) reacted poorly (at titers that were eightfold or greater reduced compared with the homologous virus titer) but were antigenically related to the previous vaccine virus B/Brisbane/60/2008 and belonged to clade V1A.

## Antiviral Susceptibility of Influenza Viruses

Testing of influenza A(H1N1)pdm09, influenza A(H3N2), and influenza B viruses for resistance to the neuraminidase inhibitors oseltamivir, zanamivir, and peramivir is performed at CDC using next generation sequencing analysis, a functional assay, or both. Neuraminidase sequences of viruses are examined for the presence of amino acid substitutions, previously associated with reduced or highly reduced inhibition by any of the three neuraminidase inhibitors.^¶^ The amino acid substitution H275Y is considered clinically relevant, because of the frequency of occurrence and the availability of clinical data to demonstrate a reduced treatment efficacy; however, the other amino acid substitutions have been observed less frequently and caused reduced susceptibility in vitro but with clinical significance being less clear (*2*).

A total of 823 influenza virus specimens (481 influenza A(H1N1)pdm09, 254 influenza A(H3N2), 34 influenza B/Victoria, and 54 influenza B/Yamagata viruses) collected in the United States during October 1, 2018–February 2, 2019, were tested for resistance to oseltamivir, zanamivir, and peramivir. Two (0.4%) influenza A(H1N1)pdm09 viruses displayed highly reduced inhibition by oseltamivir and peramivir. An additional two (0.4%) influenza A(H1N1)pdm09 viruses displayed reduced inhibition by oseltamivir. All influenza viruses tested were found to be sensitive to zanamivir. Reporting of baloxavir susceptibility testing for the 2018–19 influenza season will begin later this season. High levels of resistance to the adamantanes (amantadine and rimantadine) persist among influenza A(H1N1)pdm09 and influenza A(H3N2) viruses (the adamantanes are not effective against influenza B viruses).

## Outpatient Illness Surveillance

**FIGURE 3:**
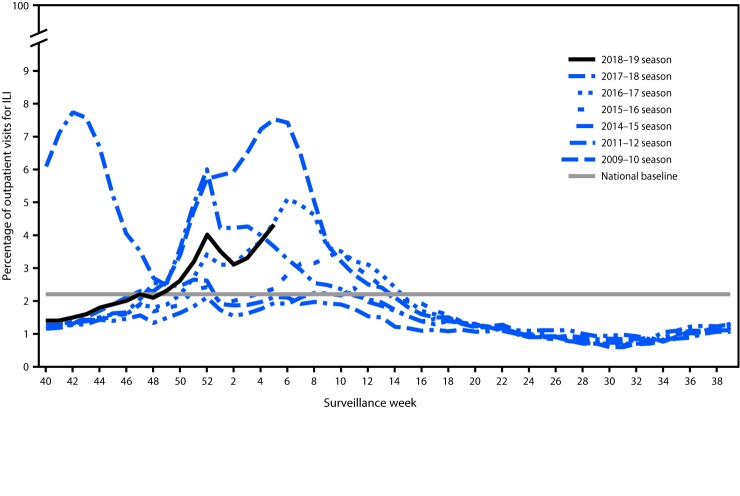
Percentage of outpatient visits for influenza-like illness (ILI)* reported to CDC, by surveillance week – U.S. Outpatient Influenza-Like Illness Surveillance Network, 2018–19 influenza season and selected previous influenza seasons^†^ * Defined as fever (temperature of ≥100°F [≥37.8°C], oral or equivalent) and cough or sore throat, without a known cause other than influenza. ^†^ As of February 2, 2019.

Nationally, during September 30, 2018–February 2, 2019, the weekly percentage of outpatient visits for ILI** to health care providers participating in the United States Outpatient Influenza-like Illness Surveillance Network (ILINet) has been at or above the national baseline^††^ level of 2.2% for 9 consecutive weeks (weeks 49–5) (Figure 3). For the week ending February 2, 2019 (week 5), the percentage of outpatient visits for ILI was 4.3%, and all 10 U.S. Department of Health and Human Services regions^§§^ reported ILI activity at or above region-specific baseline levels. During the past five influenza seasons, the peak percentage of visits for ILI has ranged from 3.6% (2015–16) to 7.5% (2017–18) and remained at or above baseline levels for an average of 16 weeks (range = 11–20 weeks).

ILINet data are used to produce a weekly jurisdiction-level measure of ILI activity,^¶¶^ ranging from minimal to high. For the weeks ending October 6, 2018–February 2, 2019, fewer than half of the 53 jurisdictions reporting to ILINet (50 states, New York City, the District of Columbia, and Puerto Rico) experienced high ILI activity each week, with the highest number (25; 47%) during the week ending February 2, 2019 (week 5). During the past five seasons, the largest number of jurisdictions experiencing high ILI activity in a single week ranged from 16 (30%) during the 2015–16 season to 46 (87%) during the 2017–18 season.

## Geographic Spread of Influenza Activity

State and territorial epidemiologists report the geographic distribution of influenza in their jurisdictions (50 states, District of Columbia, Guam, Puerto Rico, and U.S. Virgin Islands) through a weekly influenza activity code.*** During September 30, 2018–February 2, 2019, the peak number of jurisdictions reporting widespread activity in a single week was 48 (89%); this occurred during week 5 (the week ending February 2, 2019). During the previous five influenza seasons, the peak number of jurisdictions reporting widespread activity in a single week during each season has ranged from 41 (76%) (2015–16 season) to 50 (93%) (2017–18 season).

## Influenza-Associated Hospitalizations

CDC monitors hospitalizations associated with laboratory-confirmed influenza infections through the Influenza Hospitalization Surveillance Network (FluSurv-NET),^†††^ which covers approximately 27 million persons (9% of the U.S. population). In addition, FluSurv-NET data are being used to generate preliminary national estimates of the cumulative in-season numbers of symptomatic illnesses, medical visits, hospitalizations, and deaths in the United States.

During October 1, 2018–February 2, 2019, a total of 5,791 laboratory-confirmed influenza-related hospitalizations were reported (cumulative incidence for all age groups = 20.1 per 100,000 population). By age group, the cumulative hospitalization rate was 33.5 per 100,000 population among children aged 0–4 years, 7.6 among children and adolescents aged 5–17 years, 9.6 among adults aged 18–49 years, 27.2 among adults aged 50–64 years, and 53.0 among adults aged ≥65 years. Among 5,791 hospitalizations, 5,434 (93.8%) were associated with influenza A virus, 299 (5.2%) with influenza B virus, 28 (0.5%) with influenza A virus and influenza B virus co-infection, and 30 (0.5%) with an influenza virus for which the type was not determined. Among hospitalizations associated with influenza A for which subtype information was known, 975 (76.8%) were A(H1N1)pdm09 virus, and 294 (23.2%) were A(H3N2).

Complete medical chart abstraction data in FluSurv-NET will not be finalized until later in 2019; however, as of February 2, 2019, data were available for 905 (15.6%) hospitalized adults and children with laboratory-confirmed influenza. Among 755 hospitalized adults with information on underlying medical conditions,^§§§^ 681 (90.2%) had at least one reported underlying medical condition; those most commonly reported were cardiovascular disease (40.6% of 681), obesity (40.1%), and metabolic disorder (39.3%). Among 150 hospitalized children with information on underlying medical conditions, 62 (41.3%) had at least one underlying medical condition; the most commonly reported being asthma (19.7% of 150) and obesity (11.0%). Among 131 hospitalized women aged 15–44 years with information on pregnancy status, 20 (15.3%) were pregnant.

## Pneumonia and Influenza-Associated Mortality

CDC tracks pneumonia and influenza (P&I)–attributed deaths through CDC’s National Center for Health Statistics (NCHS) Mortality Reporting System. To allow for collection of sufficient data to produce stable P&I percentages, NCHS surveillance data are released 2 weeks after the week of death. During September 30, 2018–January 26, 2019, based on data from NCHS, the weekly percentage of deaths attributed to P&I ranged from 5.5% to 7.4%. P&I has been at or above the epidemic threshold^¶¶¶^ for 3 consecutive weeks (the weeks ending January 5–January 19, 2019).

## Influenza-Associated Pediatric Mortality

CDC monitors influenza-associated deaths among children aged <18 years through the Influenza-Associated Pediatric Mortality Surveillance System. As of February 2, 2019, a total of 28 laboratory-confirmed influenza-associated pediatric deaths during the 2018–19 season had been reported to CDC from New York City and 21 states. One death occurred in a non-U.S. resident. Fifteen (54%) of these deaths were associated with an infection with an influenza A(H1N1)pdm09 virus, two (7%) with an influenza A(H3N2) virus, 10 (36%) with an influenza A virus for which no subtyping was performed, and one (4%) with an influenza B virus. The mean age of the pediatric deaths reported this season was 6.5 years (range = 8 months–15 years); 15 (54%) children died after admission to the hospital. Among the 26 children who died with a known medical history, 12 (46%) had at least one underlying medical condition recognized by the Advisory Committee on Immunization Practices (ACIP) as placing them at high risk for influenza-related complications (*3*). Among the 22 children who were eligible for influenza vaccination and for whom vaccination status was known, six had received at least 1 dose of influenza vaccine before illness onset (three were fully vaccinated according to 2018 ACIP recommendations, and three had received 1 of 2 recommended doses). Since influenza-associated pediatric mortality became a nationally notifiable condition in 2004, the total number of influenza-associated pediatric deaths each season has ranged from 37 during the 2011–12 season to 185 during the 2017–18 season. These numbers are likely an underestimate of the actual number of influenza-associated pediatric deaths.

## Preliminary Prevalence Estimates of Influenza

CDC estimates the cumulative prevalence of influenza using the cumulative rates of influenza-associated hospitalizations reported through FluSurv-NET and a mathematical model.**** From October 1, 2018 to February 2, 2019, CDC estimates that influenza virus infection has caused 13,200,000–15,200,000 symptomatic illnesses, 6,170,000–7,220,000 medical visits, 155,000–186,000 hospitalizations, and 9,600–15,900 deaths.

## Discussion

In the United States, influenza activity remained elevated through early February. Influenza A(H1N1)pdm09 viruses have predominated nationwide, but influenza A(H3N2) viruses have predominated in the southeastern United States. Influenza A(H3N2) viruses have accounted for an increasing proportion of reported influenza viruses in several regions. The number of influenza B viruses reported has been low; influenza B/Yamagata viruses were more commonly reported from September through late December, and influenza B/Victoria viruses have been reported more frequently since late December. ILI activity and the percentage of respiratory specimens testing positive for influenza in clinical laboratories have been increasing since mid-January. This season, the percentage of outpatient ILI visits has reached 4.3% at the beginning of February. The peak ILI activity for the past two A(H1N1)pdm09-predominant seasons was 3.6% during the 2015–16 season and 4.6% during the 2013–14 season. Influenza-associated hospitalization rates and P&I-attributed mortality have been relatively low this season and are consistent with what has been observed during previous seasons when influenza A(H1N1)pdm09 viruses predominated (*4*,*5*). During most seasons, including this season, adults aged ≥65 years have the highest hospitalization rates, followed by children aged <5 years. Severity indicators demonstrate that, as of February 2, 2019, the severity of influenza activity has been low; however preliminary cumulative in-season prevalence estimates indicate that influenza has caused 155,000–186,000 hospitalizations and 9,600–15,900 deaths. Current influenza forecasts^††††^ predict that elevated influenza activity in parts of the United States will continue for several more weeks.

Most of the influenza viruses characterized during this time are antigenically similar to the cell culture-propagated reference viruses representing the 2018–19 Northern Hemisphere influenza vaccine viruses. However, genetic diversity among currently circulating influenza A(H1N1)pdm09 viruses belonging to clade 6B.1 viruses has increased, suggesting ongoing evolution of these viruses. Increased circulation and testing of 3C.3a viruses has contributed to a recent increasing proportion of A(H3N2) viruses that are antigenically distinct from the reference virus representing the A(H3N2) vaccine component. The majority of influenza viruses collected since October 1, 2018, and tested (>99%) displayed susceptibility to oseltamivir and peramivir, and all tested viruses displayed susceptibility to zanamivir.

The 2018–19 season is the first season that CDC has reported preliminary estimates of the prevalence of influenza in the United States during the season, and prevalence estimates will be updated each week over the remainder of the season. CDC estimates that since the 2010–11 season, during an influenza season, influenza virus infection has caused 9.3 million–49 million symptomatic illnesses, 4.3 million–23 million medical visits, 140,000–960,000 hospitalizations, and 12,000-79,000 deaths^§§§§^.

Health care providers should continue to offer and encourage vaccination to all unvaccinated persons aged ≥6 months as long as influenza viruses are circulating (*3*). Interim estimates of vaccine effectiveness based on data collected during November 23, 2018–February 2, 2019, indicate that, overall, the influenza vaccine has been 47% (95% confidence interval = 34%–57%) effective in preventing medically attended acute respiratory virus infection across all age groups and specifically was 46% (30%–58%) effective in preventing medical visits associated with influenza A(H1N1)pdm09 (*6*). Annual influenza vaccination is the first and best defense against influenza infection. Depending on the vaccine formulation (trivalent or quadrivalent), influenza vaccines can protect against three or four different influenza viruses. With vaccine effectiveness in the range of 30%–60%, influenza vaccination prevents millions of infections and medical visits and tens of thousands of influenza-associated hospitalizations each year in the United States.^¶¶¶¶^ During the 2017–18 season, vaccination averted an estimated 7.1 million illnesses, 3.7 million medical visits, 109,000 influenza-associated hospitalizations, and 8,000 influenza-associated deaths (*7*). In addition, influenza vaccination has been found to reduce deaths, intensive care unit admissions and length of stay, and overall duration of hospitalization among hospitalized influenza patients (*8*).

Influenza antiviral medications are an important adjunct to vaccination in the treatment and prevention of influenza. Treatment as soon as possible with influenza antiviral medications is recommended for patients with confirmed or suspected influenza who have severe, complicated, or progressive illness; who require hospitalization; or who are at high risk for influenza complications. Providers should not rely on less sensitive assays such as rapid antigen detection influenza diagnostic tests to inform treatment decisions (*9*). Four influenza antiviral drugs are approved by the Food and Drug Administration (FDA) for treatment of acute uncomplicated influenza within 2 days of illness onset and are recommended for use in the United States during the 2018–19 season: oseltamivir, zanamivir, peramivir, and baloxavir, which was approved by the FDA on October 24, 2018 (*10*).

Influenza surveillance reports for the United States are posted online weekly (https://www.cdc.gov/flu/weekly). Additional information regarding influenza viruses, influenza surveillance, influenza vaccine, influenza antiviral medications, and novel influenza A infections in humans is available online (https://www.cdc.gov/flu).

**Summary**

**What is already known about this topic?**

CDC collects, compiles, and analyzes data on influenza activity and viruses in the United States.

**What is added by this report?**

Influenza activity in the United States remained elevated through February 2, 2019, and is expected to continue for several more weeks. Compared with recent influenza seasons, as of February 2, 2019, severity this season has been low, with a lower percentage of outpatient visits for influenza-like illness, lower rates of hospitalization, and fewer deaths attributed to pneumonia and influenza.

**What are the implications for public health practice?**

Influenza vaccination remains the most effective way to prevent influenza illness. Influenza antiviral medications are an important adjunct to vaccination in the treatment and prevention of influenza.

## Footnotes

* Data as of February 2, 2019.

† The CDC influenza surveillance system collects five categories of information from eight data sources: 1) virus surveillance (U.S. World Health Organization collaborating laboratories, the National Respiratory and Enteric Virus Surveillance System (NREVSS), and novel influenza A virus case reporting); 2) outpatient illness surveillance (U.S. Outpatient Influenza-Like Illness Surveillance Network [ILI-Net]); 3) mortality (the National Center for Health Statistics Mortality Surveillance System and influenza-associated pediatric mortality reports); 4) hospitalizations (FluSurv-NET, which includes the Emerging Infections Program and surveillance in three additional states); and 5) summary of the geographic spread of influenza (state and territorial epidemiologist reports). https://www.cdc.gov/flu/weekly/fluactivitysurv.htm.

§ Association of Public Health Laboratories. Influenza Virologic Surveillance Right Size Roadmap. https://www.aphl.org/AboutAPHL/publications/Documents/ID_July2013_Influenza-Virologic-Surveillance-Right-Size-Roadmap.pdf.

¶ https://www.who.int/influenza/gisrs_laboratory/antiviral_susceptibility/NAI_Reduced_Susceptibility_Marker_Table_WHO.pdf?ua = 1.

** Defined as a fever (temperature ≥100°F [≥37.8°C], oral or equivalent) and cough or sore throat, without a known cause other than influenza.

†† The national and regional baselines are the mean percentages of visits for ILI during noninfluenza weeks for the previous three seasons plus two standard deviations. Noninfluenza weeks are defined as periods of ≥2 consecutive weeks during which each week accounted for <2% of the season’s total number of specimens that tested positive for influenza. National and regional percentages of patient visits for ILI are weighted based on state population. Use of the national baseline for regional data are not appropriate.

§§ The 10 U.S. Department of Health and Human Services regions include the following jurisdictions: *Region 1*: Connecticut, Maine, Massachusetts, New Hampshire, Rhode Island, and Vermont; *Region 2*: New Jersey, New York, Puerto Rico, and the U.S. Virgin Islands; *Region 3*: Delaware, District of Columbia, Maryland, Pennsylvania, Virginia, and West Virginia; *Region 4*: Alabama, Florida, Georgia, Kentucky, Mississippi, North Carolina, South Carolina, and Tennessee; *Region 5*: Illinois, Indiana, Michigan, Minnesota, Ohio, and Wisconsin; *Region 6*: Arkansas, Louisiana, New Mexico, Oklahoma, and Texas; *Region 7*: Iowa, Kansas, Missouri, and Nebraska; *Region 8*: Colorado, Montana, North Dakota, South Dakota, Utah, and Wyoming; *Region 9*: Arizona, California, Hawaii, Nevada, American Samoa, Commonwealth of the Northern Mariana Islands, Federated States of Micronesia, Guam, Marshall Islands, and Republic of Palau; *Region 10*: Alaska, Idaho, Oregon, and Washington.

¶¶ Activity levels are based on the percentage of outpatient visits in a jurisdiction attributed to ILI and are compared with the average percentage of ILI visits that occur during weeks with little or no influenza virus circulation. Activity levels range from minimal (corresponding to ILI activity from outpatient clinics at or below the average) to high (corresponding to ILI activity from outpatient clinics much higher than the average). Because the clinical definition of ILI is nonspecific, not all ILI is caused by influenza; however, when combined with laboratory data, the information on ILI activity provides a clearer picture of influenza activity in the United States.

*** Levels of activity are 1) no activity; 2) sporadic: isolated laboratory-confirmed influenza cases or a laboratory-confirmed outbreak in one institution, with no increase in activity; 3) local: increased ILI, or two or more institutional outbreaks (ILI or laboratory-confirmed influenza) in one region of the state, with recent laboratory evidence of influenza in that region; virus activity no greater than sporadic in other regions; 4) regional: increased ILI activity or institutional outbreaks (ILI or laboratory-confirmed influenza) in two or more outbreaks, but with fewer than half of the regions in the state with recent laboratory evidence of influenza in those regions; and 5) widespread: increased ILI activity or institutional outbreaks (ILI or laboratory-confirmed influenza) in at least half of the regions in the state, with recent laboratory evidence of influenza in the state.

††† FluSurv-NET conducts population-based surveillance for laboratory-confirmed, influenza-associated hospitalizations in children and adolescents aged <18 years (since the 2003–04 influenza season) and adults aged ≥18 years (since the 2005–06 influenza season). FluSurv-NET covers approximately 70 counties in the 10 states in the Emerging Infections Program (California, Colorado, Connecticut, Georgia, Maryland, Minnesota, New Mexico, New York, Oregon, and Tennessee) and additional Influenza Hospitalization Surveillance Project (IHSP) states. IHSP began during the 2009–10 season to enhance surveillance during the 2009 H1N1 pandemic. IHSP sites included Idaho, Iowa, Michigan, Oklahoma, and South Dakota during the 2009–10 season; Idaho, Michigan, Ohio, Oklahoma, Rhode Island, and Utah during the 2010–11 season; Michigan, Ohio, Rhode Island, and Utah during the 2011–12 season; Iowa, Michigan, Ohio, Rhode Island, and Utah during the 2012–13 season; and Michigan, Ohio, and Utah during the 2013–14, 2014–15, 2015–16, 2016–17, 2017–18, and 2018–19 seasons. Cumulative unadjusted incidence rates are calculated using CDC’s National Center for Health Statistics population estimates for the counties included in the surveillance catchment area. Laboratory confirmation is dependent on clinician-ordered influenza testing and testing for influenza often is underutilized because of the poor reliability of rapid test results and greater reliance on clinical diagnosis for influenza. Therefore, cases identified as part of influenza hospitalization surveillance likely are an underestimation of the actual number of persons hospitalized with influenza.

§§§ Persons at higher risk include 1) children aged <2 years; 2) adults aged ≥65 years; 3) persons with chronic pulmonary conditions (including asthma), cardiovascular disease (except hypertension alone), renal, hepatic, hematologic (including sickle cell) disease, metabolic disorders (including diabetes mellitus), or neurologic and neurodevelopmental conditions (including disorders of the brain, spinal cord, peripheral nerves, and muscles, such as cerebral palsy, epilepsy [seizure disorders], stroke, intellectual disability [mental retardation], moderate to severe developmental delay, muscular dystrophy, or spinal cord injury); 4) persons with immunosuppression, including that caused by medications or by human immunodeficiency virus infection; 5) women who are pregnant or postpartum (within 2 weeks after delivery); 6) persons aged ≤18 years who are receiving long-term aspirin therapy; 7) American Indians/Alaska Natives; 8) persons with extreme obesity (i.e., body mass index ≥40); and 9) residents of nursing homes and other chronic care facilities.

¶¶¶ The seasonal baseline proportion of P&I deaths is projected using a robust regression procedure, in which a periodic regression model is applied to the observed percentage of deaths from P&I that were reported by the National Center for Health Statistics Mortality Surveillance System during the preceding 5 years. The epidemic threshold is set at 1.645 standard deviations above the seasonal baseline.

**** https://www.cdc.gov/flu/about/burden/how-cdc-estimates.htm.

†††† https://www.cdc.gov/flu/weekly/flusight/index.html.

§§§§
https://www.cdc.gov/flu/about/burden/past-seasons.html .

¶¶¶¶ https://www.cdc.gov/flu/about/disease/2016-17.htm.
